# Metagenomic study of food waste anaerobic digestion

**DOI:** 10.1128/spectrum.02087-25

**Published:** 2025-10-22

**Authors:** Oluwatomisin A. Akinsola, Samuel O. Dahunsi, Ebenezer L. Odekanle

**Affiliations:** 1 Microbiology Programme, College of Agriculture, Engineering, and Sciences, Bowen University 70671, Iwo, Osun State, Nigeria; 2 The Radcliffe Institute for Advanced Study, Harvard University 1812, Cambridge, Massachusetts, USA; 3 Department of Chemical and Petroleum Engineering, First Technical University 532812, Ibadan, Oyo State, Nigeria; Ruhr-Universitat Bochum, Bochum, Germany

**Keywords:** affordable and clean energy, anaerobic digestion, cleaner production, circular bioeconomy, metagenomic analysis, microbial taxonomy

## Abstract

This study explores anaerobic digestion of food waste to understand the microbial community dynamics and metabolic pathways that drive the conversion of organic waste into biogas. Sampling was done at multiple time points during those 4 weeks (weekly) to capture microbial succession/changes over time. The microbial profile was evaluated using QIIME2 and BV-BRC, while functional annotation tools (PICRUSt2) were used to identify dominant pathways. The results reveal a temporal shift in microbial communities, with fermentative bacteria, such as *Lactobacillus* and *Clostridia,* dominating the early stages of digestion, followed by methanogenic archaea like *Methanomicrobia* in the later stages. Pathway analysis showed that fermentation, aromatic compound degradation, and methanogenesis were the primary metabolic processes, with methanogenesis becoming more prominent by week 3 (FW3_S162_R1). The study highlights the critical role of microbial community adaptation in maximizing methane production and offers new insights into optimizing anaerobic digestion for more efficient food waste biogas generation. By combining metagenomic and metabolomic approaches, this research provides a comprehensive understanding of the microbial and metabolic factors that shape the anaerobic digestion process, contributing to the development of sustainable waste management practices.

**IMPORTANCE:**

This study employs a metagenomic approach to elucidate the intricate microbial communities and metabolic processes involved in the anaerobic digestion of food waste. It highlights microbial interactions that influence biogas production, offering insights for optimizing waste-to-energy conversion. Understanding these dynamics is key to improving digestion efficiency, reducing environmental impacts, and advancing sustainable waste management and circular economy strategies. The findings provide a valuable foundation for future innovations addressing global waste and energy challenges.

## INTRODUCTION

The increasing global generation of food waste has heightened concerns about its environmental and economic impacts, driving the need for innovative and sustainable strategies to mitigate it (1). Among the various waste management technologies, anaerobic digestion (AD) has emerged as a promising method for food waste management, offering a two-sided benefit, which is the production of biogas and the reduction of waste volume (2). However, the efficiency of AD relies on the activity of complex microbial communities that break down organic materials in the absence of oxygen, ultimately converting them into methane and other by-products. These microbial communities, consisting of bacteria, archaea, and fungi, play crucial roles in the degradation of food waste, making their study vital for enhancing the efficiency of the AD process (3, 4).

The success of AD is dependent on the microbial composition and the interactions that exist within the reactors. The structural and functional diversity of these microbial communities has become a focal point of research, as their activity is influenced by the composition of the waste and the operating conditions of the digesters (5). Recent advancements in metagenomic sequencing technologies have revolutionized the ability to explore the taxonomic and metabolic profiles of these microbial communities with unprecedented resolution, offering insights into the functional roles of microorganisms in the digestion process (3, 4). These technologies have enabled researchers to identify key microbial taxa, their interactions, and metabolic pathways, which are crucial for optimizing biogas production and improving the overall efficiency of food waste digestion systems.

A diverse range of microorganisms, including bacteria and archaea, participates in the various stages of AD. Studies have shown that bacterial phyla, such as *Firmicutes* and *Bacteroidetes,* dominate the microbial community in food waste digesters, playing critical roles in hydrolysis and acidogenesis (6, 7). Archaea, particularly methanogens from the *Methanobacteria* and *Methanomicrobia* classes, are vital for methane production, catalyzing the final step in the anaerobic degradation process (8). Metagenomic analysis has uncovered specific metabolic pathways that facilitate the breakdown of various organic compounds, including lipids, proteins, and carbohydrates, in food waste (9). These insights have paved the way for developing strategies that enhance microbial efficiency in AD systems, leading to improved biogas yields and better resource recovery (10, 11).

While taxonomic diversity provides a foundational understanding of the microbial players involved, the metabolic potential of these communities is equally important in determining the efficiency of food waste digestion. Volatile fatty acids (VFAs), which are intermediate metabolites produced during digestion, significantly influence the production of methane and other biogas components. The pathways that microorganisms use to convert complex organic substrates into VFAs and methane can vary based on the composition of the food waste feedstock and the operational conditions of the digester (10, 11). For instance, certain microbial taxa are specialized in breaking down carbohydrates, while others focus on the degradation of lipids or proteins, thereby contributing to the overall efficiency of the digestion process. The interplay between these different microbial groups ensures a continuous flow of metabolites, ultimately leading to the production of biogas. Understanding these metabolic pathways allows researchers to devise strategies for optimizing methane production, such as adjusting operating conditions to promote the activity of specific microbial groups (3, 4). Additionally, the application of high-throughput sequencing technologies has enabled the identification of microbial taxa that specialize in specific metabolic functions, such as the degradation of cellulose, lipids, or proteins, providing new opportunities for optimizing the AD process (3, 4).

The implications of these findings extend beyond the biogas production process itself. By improving the efficiency of food waste digestion, the recovery of nutrients from the digestate (the solid by-product of AD) can support sustainable agricultural practices, such as fertilization, thereby promoting the principles of the circular economy (12, 13). Furthermore, anaerobic digestion plays a critical role in mitigating the environmental impacts of food waste disposal, such as greenhouse gas emissions and landfill space usage (14, 15). As such, understanding the microbial communities involved in food waste digestion can have far-reaching environmental, economic, and policy implications.

Despite the significant progress in the characterization of microbial communities in AD systems, there remain notable gaps in their understanding, particularly in the comparative analysis of microbial communities from different food waste substrates and operational conditions. The taxonomic and metabolic diversity of microbial populations under varied conditions remains underexplored, and more research is needed to determine the optimal conditions for specific microbial groups to thrive. Additionally, there is a lack of detailed studies comparing the microbial community structures and their associated metabolic pathways across different food waste types. This study, therefore, aims to address some of these gaps by conducting a comprehensive metagenomic analysis of microbial communities involved in food waste anaerobic digestion. Specifically, this study focuses on identifying the taxonomic diversity and metabolic pathways associated with food waste digestion, exploring the impact of food waste substrate on microbial efficiency, and developing insights that can be used to optimize biogas production and resource recovery.

This study provides a comprehensive analysis of the microbial communities involved in food waste anaerobic digestion. By examining the taxonomic and metabolic diversity of these communities, the study will contribute to the development of targeted strategies for improving biogas production and advancing the sustainable management of food waste. In doing so, this study aims to support the optimization of anaerobic digestion processes and provide valuable insights for the design of more efficient, environmentally friendly waste management systems.

## MATERIALS AND METHODS

Food waste was collected from various cafeterias (consisting of leftover rice, beans, yams, spaghetti, meats, fried plantains, boiled eggs, and fish bones) in Bowen University, Iwo, Osun State, southwestern Nigeria. They were collected in new Ziplock bags and kept in ice coolers to avoid further deterioration while being transported to the laboratory, where the samples were kept in the refrigerator before further analysis. The physicochemical and nutrient composition of food waste is shown in Table 1.

**TABLE 1:** Physicochemical and nutrient composition of food waste used in the digestion^*a*^

Parameters	Food waste
pH	6.50 ± 0.02
Total nitrogen (mg/L)	26.41 ± 2.01
Total phosphorus (mg/L)	4.21 ± 0.02
Total carbon (mg/L)	255.11 ± 10.02
Potassium (mg/L)	5.11 ± 0.04
Phosphate (mg/L)	115.81 ± 8.02
Sulfate (mg/L)	76.22 ± 6.12
Calcium (mg/L)	78.41 ± 7.04
Magnesium (mg/L)	46.01 ± 4.12
Manganese (mg/L)	0.014 ± 0.02
Iron (mg/L)	3.96 ± 0.21
Zinc (mg/L)	14.61 ± 1.02
Aluminum (mg/L)	0.48 ± 0.01
Copper (mg/L)	3.24 ± 0.21
COD (g COD/g VS)	118.41 ± 7.02
C/N ratio	10/1
Ammonia (mg/L)	4.21 ± 0.30
Cellulose (% VS)	16.12 ± 2.12
Hemicelluloses (% VS)	9.32 ± 1.02
Klason lignin (% VS)	17.52 ± 2.05
Uronic acids (% VS)	0.64 ± 0.20
^@^Soluble sugars (% VS)	8.49 ± 1.02
Phenols (mg/L)	0.64 ± 0.21
TVFAs (g COD/g VS)	0.33 ± 0.02
Moisture content (%)	77.82 ± 5.02
Total solids (%)	22.77 ± 2.03
Ash content (%)	18.98 ± 1.07
Volatile solids (%)	79.53 ± 6.03

^*a*^ COD, chemical oxygen demand; C/N, carbon/nitrogen ratio; TVFAs, total volatile fatty acids; @, sum of initial soluble sugars and the solubilization of cellulose and hemicelluloses.

### Description of bioreactor

In this study, the computer-controlled batch anaerobic bioreactor (EDIBON, United Kingdom) was utilized. The bioreactor is comprised of two double-jacketed anaerobic chambers, each with a capacity of 10 L. These chambers are equipped to monitor and regulate digestion parameters, including pH levels, water flow, temperature, and measure gas production. The anaerobic chambers were maintained in an anoxic environment with auto-stirring, which facilitates the mixing of substrates and ensures an even distribution of microorganisms within the tanks.

### Experimental procedures for anaerobic digestion

Food waste was mixed with water and blended to form a slurry (13, 16, 17) and was loaded into each reactor to occupy 4/5 of the total volume, thereby allowing the remaining 1/5 of the volume for pressure build-up throughout the experiment. Poultry dropping was used as inoculum for the process. Also, the biogas volume produced was determined every 24 h interval by downward displacement as previously described (13). Furthermore, to analyze the composition and characteristics of the biogas produced, a gas chromatography-mass spectrometry/electron ionization system equipped with a flame ionization detector was employed.

### Microbial community analysis

To observe the variations in the microbial community structure throughout the digestion process and its performance, 5 mL of samples (weeks 1 to 4) were taken from effluents of the digestion process weekly and refrigerated at −20°C. Genomic DNA was extracted using a standardized commercial kit.

### Genomic DNA extraction

The preparation of samples for DNA extraction was conducted using Zymo Research’s Quick-16S kit, adhering to the manufacturer’s instructions. To assess the quality of the extracted DNA, both its concentration and purity were evaluated using 1% agarose gel electrophoresis. The samples were then diluted with sterile water to achieve a standardized final concentration of 1 ng/µL, ensuring uniformity across all samples for subsequent analysis (18).

### DNA extraction method

In SeqCenter, routine DNA extraction was conducted following the standardized protocols outlined in the ZymoBIOMICS DNA Miniprep Kit2 p. The samples were homogenized properly, and 200 µL of the sample was aliquoted into 550 µL of lysis solution to initiate the extraction process. The suspended cells within the lysis solution were carefully transferred into the ZR BashingBead Lysis Tubes, where they undergo mechanical disruption. This lysis procedure is carried out using the MP FastPrep-24 lysis system, which involves a 1 min lysis at maximum speed, followed by a 5 min resting phase, repeated for a total of five cycles to ensure efficient cell breakdown. Following lysis, the samples were subjected to centrifugation at 10,000 rcf for 1 min to separate the lysate from debris. From the resulting supernatant, 400 µL was transferred from the ZR BashingBead Lysis Tube to a Zymo-Spin III-F Filter and centrifuged at 8,000 rcf for 1 min. Subsequently, 1,200 µL of ZymoBIOMICS DNA Binding (19) Buffer was introduced into the collected effluent and thoroughly mixed using a pipette to ensure uniform distribution. To facilitate DNA binding, 800 µL of this mixture was loaded onto a Zymo-Spin IICR Column and centrifuged at 10,000 rcf for 1 min. This loading and centrifugation process was repeated until the entire sample was processed through the Zymo-Spin IICR Column, ensuring optimal DNA recovery and purification. To ensure optimal purity, the DNA bound to the Zymo-Spin IICR Column underwent a washing process to ensure optimal purity. This involved three sequential washes: first with 400 µL ZymoBIOMICS DNA Wash Buffer 1, followed by 700 µL of the same buffer, then 200 µL of ZymoBIOMICS DNA Wash Buffer 2. Each step was carried out with a 1 min spin down at 10,000 rcf, respectively. The purified DNA was eluted by adding 75 µL of ZymoBIOMICS DNase/RNase Free Water. This elution step was enhanced by allowing the sample to incubate at room temperature for 5 min before subjecting it to final centrifugation at 10,000 rcf for 1 min to collect the eluted DNA. To further refine the purity of the extracted DNA, the Zymo-Spin III-HRC Filter was prepared by adding 600 µL of the ZymoBIOMICS HRC Prep Solution, followed by centrifugation at 8,000 rcf for 3 min. The eluted DNA was then passed through the prepared Zymo-Spin III-HRC Filter for final purification (20). Finally, the concentration of the purified DNA was determined using Qubit 3 fluorometer.

### 16S preparation and sequencing methods

Extracted DNA samples were first quantified using a Qubit 3 fluorometer (Thermo Fisher Scientific) to assess concentration and purity. The V3/V4 regions of the 16S rRNA gene were then amplified using phased primers from Zymo Research’s Quick-16S kit, with the exact primer sequences detailed in Table 2. The PCR reaction was conducted with the following conditions: 95°C for 3 min; 25 cycles of (95°C for 30 s, 55°C for 30 s, 72°C for 30 s); followed by a final extension at 72°C for 5 min. The amplicons were purified using AMPure XP beads and eluted in 27.5 µL of 10 mM Tris, pH 8.5. The libraries were quantified, normalized, and pooled. Following clean-up and normalization, samples were sequenced using a P1 600cyc NextSeq2000 Flowcell to generate 2 × 301 bp paired-end reads. Bcl-convert1 (v4.2.4) was used to carry out quality control measures, and adapter trimming was conducted using bcl-convert1 (v4.2.4). The PCR artifacts, such as primer-dimers, were filtered based on read length (>150 bp), polyN strings (<10 Ns), and polyG strings (<150 Gs). A sampling depth of 1,000 reads was used for diversity analyses to ensure comparability across samples.

**TABLE 2:** Specific primer sequences used

Region	Forward sequence: 341f	Reverse sequence: 806r
V3/V4	CCTACGGGDGGCWGCAG CCTAYGGGGYGCWGCAG	GACTACNVGGGTMTCTAATCC

### 16S rRNA gene sequence analysis

Quality control procedures, including adapter trimming, were carried out using bcl-convert (bcl-convert, 2023) to ensure high-quality sequencing data. The processed sequences were then imported into Qiime2 (21) for comprehensive analysis. Within Qiime2, primer sequences were removed using the cutadapt plugin (22). To enhance sequence accuracy, denoising was carried out using the dada2 plugin (23), which effectively identified and corrected errors within the sequences. The cleaned sequences were then classified into operational taxonomic units (OTUs) by utilizing the Silva 138 99% OTUs full-length sequence database in combination with the VSEARCH tool, both of which were executed through Qiime2’s feature-classifier plugin (24). The resulting OTUs were then assigned to their most precise taxonomic classifications, and their abundance was normalized to show their relative frequency within each sample. To assess microbial diversity, both alpha and beta diversity analyses were conducted using the q2-diversity plugin in Qiime2 (https://qiime2.org). Additional downstream analysis was performed using BV-BRC and Nephele: microbiome analysis for microbial diversity (25). For pathway prediction, the metabolic pathways were inferred through functional profiling of metagenomic data using the NIAID Nephele pipeline, which annotates genes against the Kyoto Encyclopedia of Genes and Genomes and MetaCyc databases. Pathway abundance and dominance were determined based on normalized gene counts assigned to each pathway, allowing ranking by relative abundance.

## RESULTS AND DISCUSSION

Table 3 shows the data for biogas production from the AD of food waste. There is a steady increase in cumulative gas production over the 28-day retention time, which is characteristic of anaerobic digesters as the microbial community matures. On day 1, biogas production is zero, which reflects the initial establishment of the microbial community. The microbial groups at this stage are not yet fully active or established. As production increases from days 2 to 10, there is an increase in Firmicutes (Bacilli) and Proteobacteria populations, suggesting that microbial communities are becoming more active in metabolizing the available organic matter. From day 12, gas production begins to show more consistent increases, indicating that microbial communities have become well-established, with dominant groups supporting fermentation and methane production. A decrease in gas production after day 21 reflects changes in the microbial community and organic matter availability. The total gas production of 0.93 L by day 28 is within the expected range for food waste digestion, as earlier reported (26). The methane content in the biogas, i.e., 53.46% as shown in Table 4, is an indicator of efficient methanogenesis. This result is consistent with other studies, where methane typically makes up about 50%–60% of the biogas produced in food waste digesters (27). The other gases produced, which include carbon dioxide (25.78%), ammonia (2.17%), and hydrogen sulfide (1.04%), also provide some insight into the metabolic processes occurring during the digestion process. For example, the presence of CO₂ indicates that acetoclastic methanogenesis occurred, showing that acetate was converted into methane and carbon dioxide. Ammonia, a product of protein breakdown, and hydrogen sulfide, a result of sulfur-reducing bacteria metabolizing sulfur-containing compounds, are also commonly observed in food waste digestion (28). The presence of these gases is expected and reflects the ongoing biochemical transformations that happen in the digester.

**TABLE 3:** Results of food waste biogas production for pH 7 and temperature 43°C with hydraulic retention time of 28 days

No of days	Volume of water at daily gas production (mL)	Output per day (mm)	Production per day (L)	Cumulative gas produced
1	280	0	0	0
2	279	1	0.01628	0.01628
3	278	1	0.01628	0.03256
4	276	2	0.03256	0.06512
5	274	2	0.03256	0.09768
6	272	2	0.03256	0.13024
7	270	2	0.03256	0.1628
8	268	2	0.03256	0.19536
9	266	2	0.03256	0.22792
10	264	2	0.03256	0.26048
11	261	3	0.04884	0.30932
12	258	3	0.04884	0.35816
13	255	3	0.04884	0.407
14	253	2	0.03256	0.43956
15	250	3	0.04884	0.4884
16	248	2	0.03256	0.52096
17	245	3	0.04884	0.5698
18	243	2	0.03256	0.60236
19	241	2	0.03256	0.63492
20	238	3	0.04884	0.68376
21	236	2	0.03256	0.71632
22	234	2	0.03256	0.74888
23	232	2	0.03256	0.78144
24	230	2	0.03256	0.814
25	228	2	0.03256	0.84656
26	226	2	0.03256	0.87912
27	225	1	0.01628	0.8954
28	223	2	0.03256	0.92796

**TABLE 4:** Food waste digestion biogas composition

RT (min)	Names of gases	Molecular formula	Molecular mass	Peak area	% composition
19.3	Ethane	C_2_H_6_	30	7.18	7.17
23.4	Oxygen	O_2_	32	3.80	3.79
26.7	Carbon dioxide	CO_2_	44	25.83	25.78
28.8	Nitrogen	N_2_	28	2.37	2.37
36.2	Methane	CH_4_	16	53.56	53.46
25.9	Carbon monoxide	CO	28	3.42	3.41
5.8	Ammonia	NH_3_	20	2.17	2.17
17.8	Hydrogen	H_2_	2	1.82	0.82
39.0	Hydrogen sulfide	H_2_S	34	1.24	1.04

**Fig 1:**
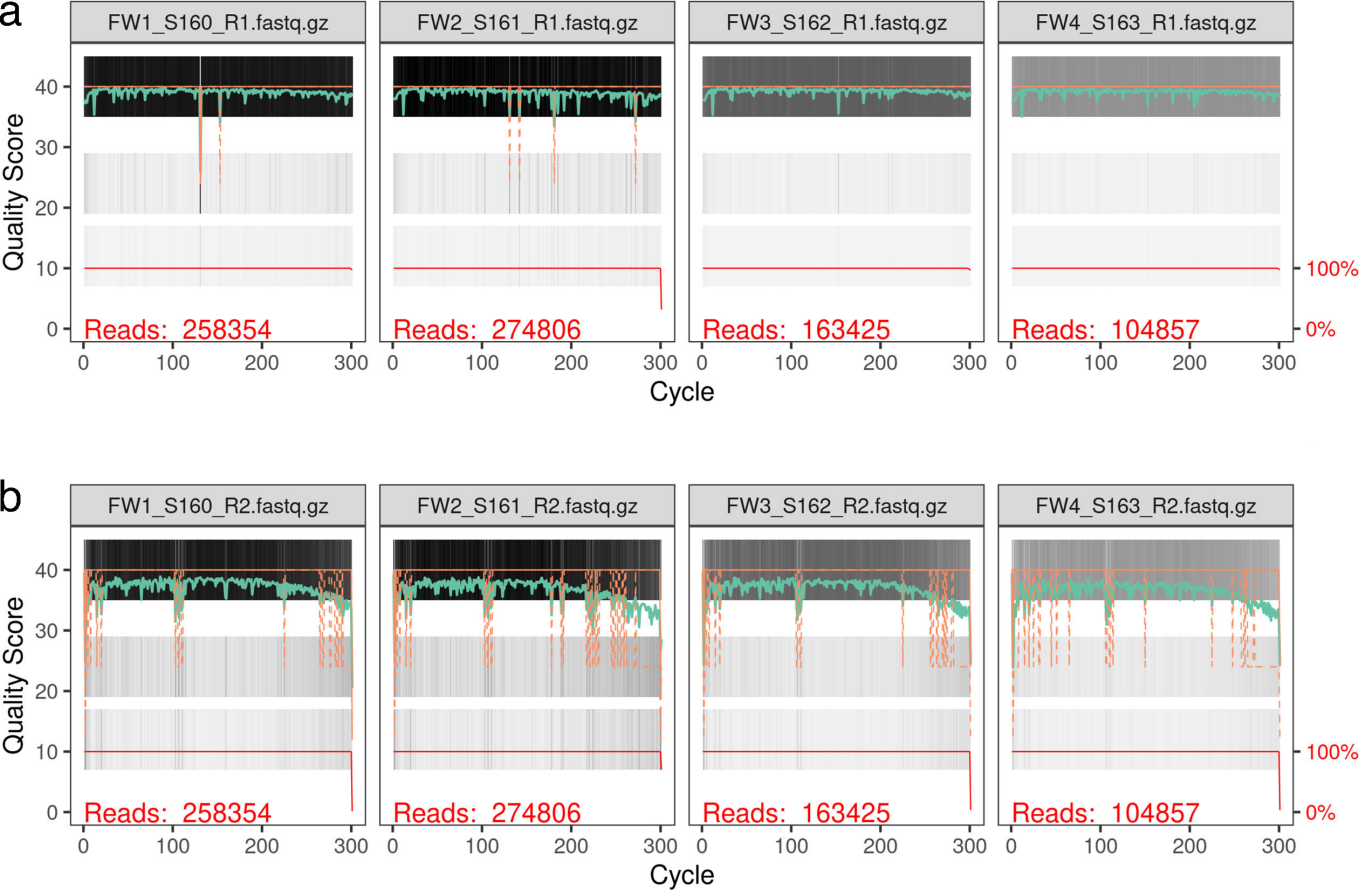
(**a**) The quality score plot for the forward reads. (**b**) The quality score plot for the reverse reads.

The quality control plots shown in Fig. 1a and b offer important insights into the integrity of the sequencing data from different time points over 4 weeks, labeled as FW1, FW2, FW3, and FW4. These plots, generated through FastQC, help assess the sequencing process’s overall performance by examining factors such as read quality, cycle-wise quality scores, and the distribution of reads across various cycles. The quality scores in the provided figures are represented by the green and cyan lines, which reflect the average Phred score across the sequencing cycles. In these plots, the quality scores are generally high in the initial cycles of sequencing (Phred score >30, corresponding to >99.9% accuracy), which is expected in most sequencing runs. The early cycles typically exhibit higher quality, as the sequencing platform starts with high precision but faces degradation in performance in later cycles due to various factors, such as signal decay or substrate limitations. As seen in the plots, there is a noticeable decline in quality scores as the sequencing progresses. In the later cycles, the quality scores drop below 20 (approximately 90% accuracy), which is a common observation in high-throughput sequencing (29). This decrease in quality suggests a higher error rate in the final cycles (30).

**Fig 2:**
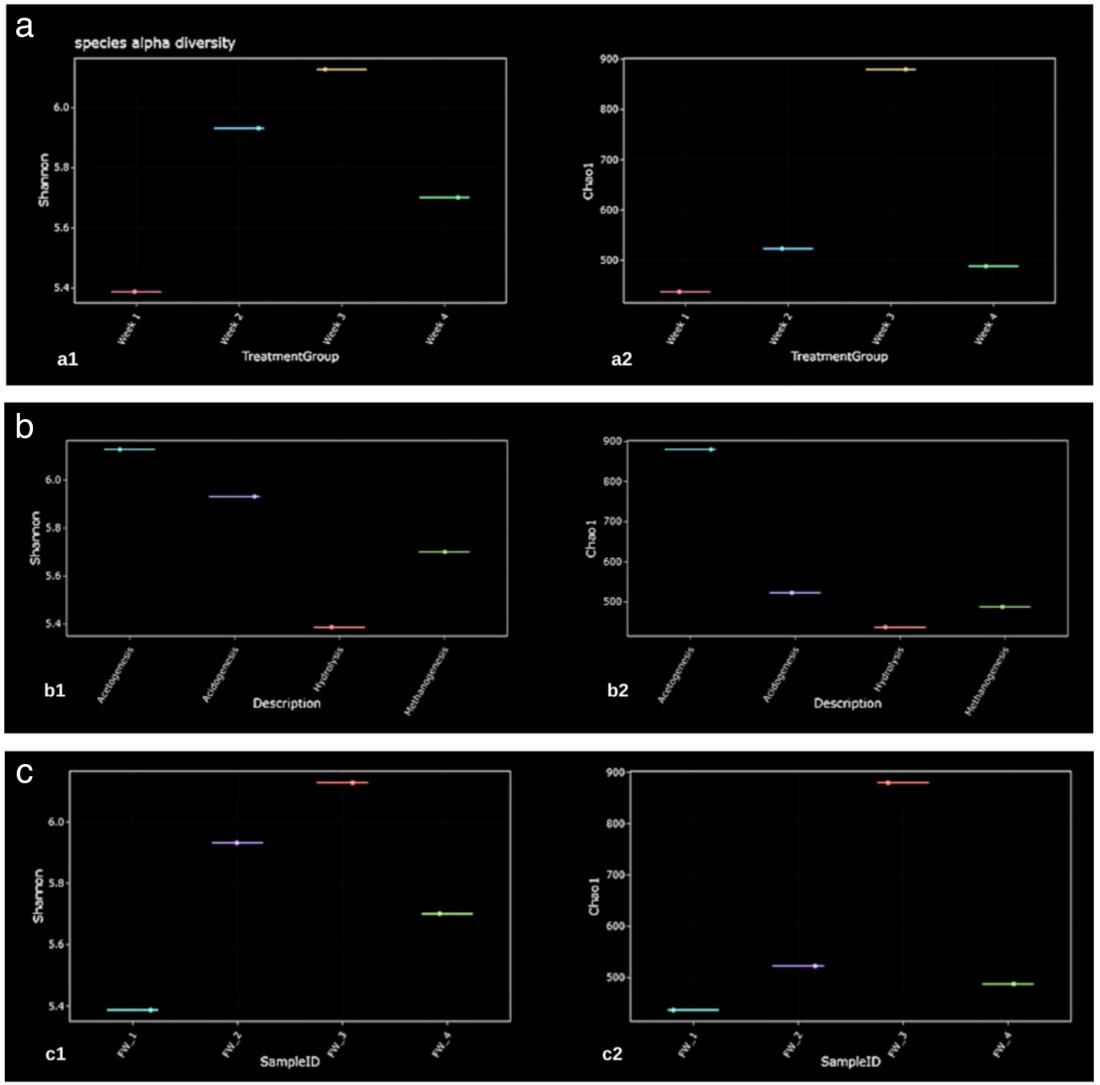
(**a–c**) The species alpha diversity is showing variations in microbial diversity across the 4 weeks.

The species alpha diversity analysis shown in Fig. 2, specifically the Shannon and Chao1 indices, shows variations in microbial diversity across the 4 weeks of the digestion process. Both Shannon diversity and Chao1 richness were highest in weeks 1 and 2, suggesting that the microbial community was more diverse and evenly distributed early on. This is typical of the initial stages of anaerobic digestion, where a variety of microbes are involved in the breakdown of the complex organic matter present in food waste. By weeks 3 and 4, however, both indices show a decrease in diversity, reflecting a shift toward more specialized microbial communities. The pattern of the diversity indices is consistent with findings from similar studies (31). Previous research on anaerobic digestion of food waste shows that microbial diversity tends to decrease as the process progresses. This is likely due to the fact that, over time, the more generalist microbes are outcompeted by those that are better suited for the remaining substrates (32). For instance, methanogens and other specialized degraders start to dominate as the digestible compounds are broken down.

**Fig 3:**
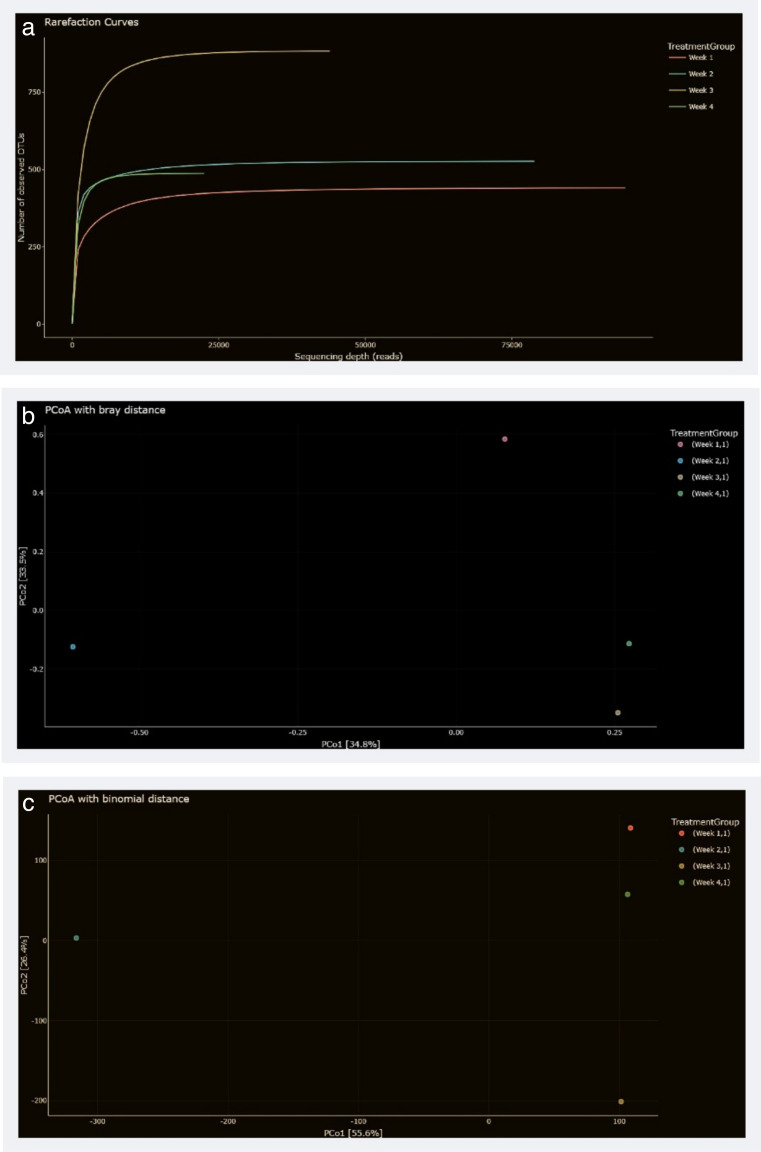
(**a**) The rarefaction curves, showing the sequencing depth and its effect on the observed number of OTUs. (**b**) The principal coordinate analysis (PCoA) plot using the Bray-Curtis distance metric. (**c**) The PCoA plot using the Binomial distance metric.

All samples were processed using standardized sequencing protocols and quality control procedures to ensure consistency across all time points. The rarefaction curves presented in Fig. 3a illustrate that as the sequencing depth increased, the number of observed OTUs also increased. In weeks 1 and 2, OTU accumulation was more gradual, reflecting ongoing microbial succession in the reactors. By week 4, the curve flattened, indicating that the sequencing effort was sufficient to capture the full microbial diversity present at that stage. This pattern is expected, as microbial communities in anaerobic digestion systems typically stabilize after a period, resulting in the full representation of key taxa by week 4. Quality control measures, including the removal of low-quality reads and chimeras, were applied before subsequent microbial community analysis, ensuring reliable diversity and composition assessment. These results are similar to those seen by reference 33, who also found that food waste digestion reaches a diversity plateau after a few weeks, and further sequencing does not significantly increase the observed microbial richness. This further supports the idea that the sequencing efforts were adequate and that the microbial communities in the samples were well captured.

The principal coordinate analysis (PCoA) plots, with Bray-Curtis and Binomial distances shown in Fig. 3b and c, provide a clear picture of how the microbial communities evolved over the 4 weeks. In the Bray-Curtis PCoA plot, week 1 is distinct from weeks 2, 3, and 4, which suggests that there was a significant shift in community composition during the first week. The closer proximity of weeks 2–4 in the plot indicates that microbial communities became more similar as the process continued, possibly due to the depletion of easily digestible organic material and the rise of more specialized organisms that can handle the residual substrates. The Binomial distance-based PCoA plot shows a similar trend. There is a separation between week 1 and the later weeks, but the grouping between weeks 2 and 4 is less pronounced, which suggests a more gradual shift in microbial communities. It shows that after the initial stage of digestion, the microbial composition in subsequent weeks became less variable, likely reflecting a more stable community. These different clustering patterns highlight how distinct distance metrics capture varied aspects of microbial community dynamics, with Bray-Curtis emphasizing abundance differences and Binomial focusing on taxa presence or absence.

These shifts align with previous studies where the microbial communities in anaerobic digestion systems tend to stabilize after an initial phase of adaptation (34). The changes observed in microbial composition over time are likely driven by the microbial community’s adaptation to the available substrates, with more specialized microbes taking over as the process progresses.

**Fig 4:**
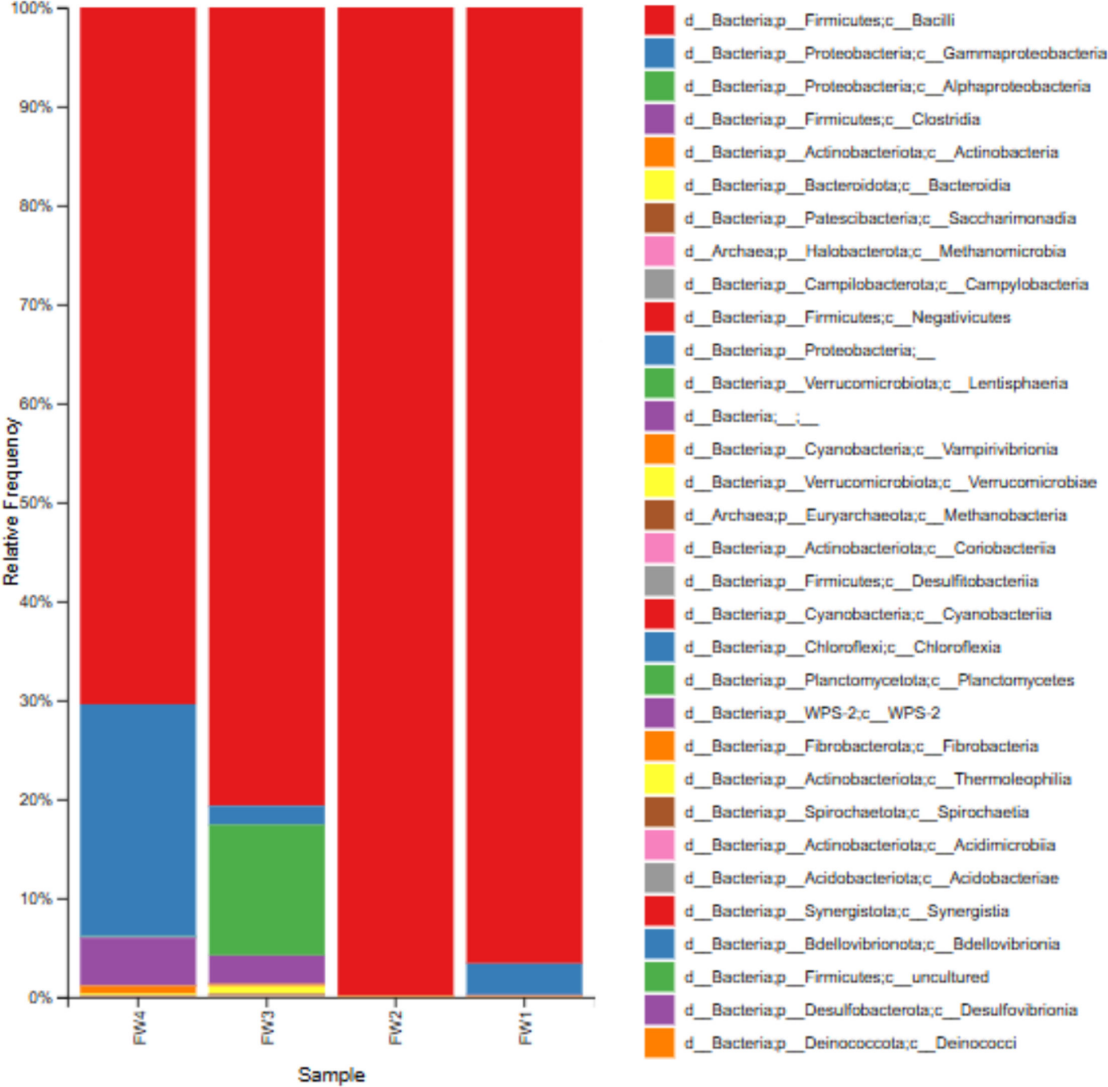
The bacterial community structure at the domain and phylum level in each sample during the course of the 4 weeks of digestion.

The metagenomic analysis of microbial communities in the AD of food waste reveals key shifts in microbial populations over time (Fig. 4). For example, bacilli (phylum Firmicutes) dominated the initial stage (FW1), while stages (FW3 and FW4) showed an increase in Proteobacteria, particularly Gammaproteobacteria and Alphaproteobacteria. As the more readily degradable substrates were consumed, specialized taxa that adapted to the residual organic matter became increasingly important. These community shifts explain the variations in biogas production observed at different stages.

In addition to these bacteria, the analysis also reveals the presence of methanogenic archaea, crucial for the final stages of anaerobic digestion, where methane is produced as a key biogas component.

In the early stages of digestion (FW1_S160_R1), *Firmicutes* dominated, which is consistent with previous studies where *Firmicutes* are recognized for their ability to ferment complex carbohydrates, such as cellulose and hemicellulose (35). *Bacteroidetes* were also prominent, particularly in FW3_S162_R1 and FW4_S163_R1, where they contributed to the breakdown of polysaccharides and proteins into VFAs (36). Actinobacteria increased from about 23,000 reads in FW1_S160_R1 to 730,000 reads in FW4_S163_R1, remaining fairly stable in other samples. Proteobacteria significantly increased from unnoticeable levels in FW1_S160_R1 to 628,000 reads in FW4_S163_R1. The increase in these phyla during later digestion stages reflects their role in breaking down complex organics into simpler molecules that enhance biogas production (37). The presence *of Lactobacillaceae* in multiple samples indicates active lactic acid fermentation, which contributes to acidogenesis in anaerobic digestion (38). The appearance of *Pseudomonadaceae* and *Weissella* in later samples highlights their role in the degradation of aromatic compounds, which are generally more resistant to anaerobic digestion (38).

**Fig 5:**
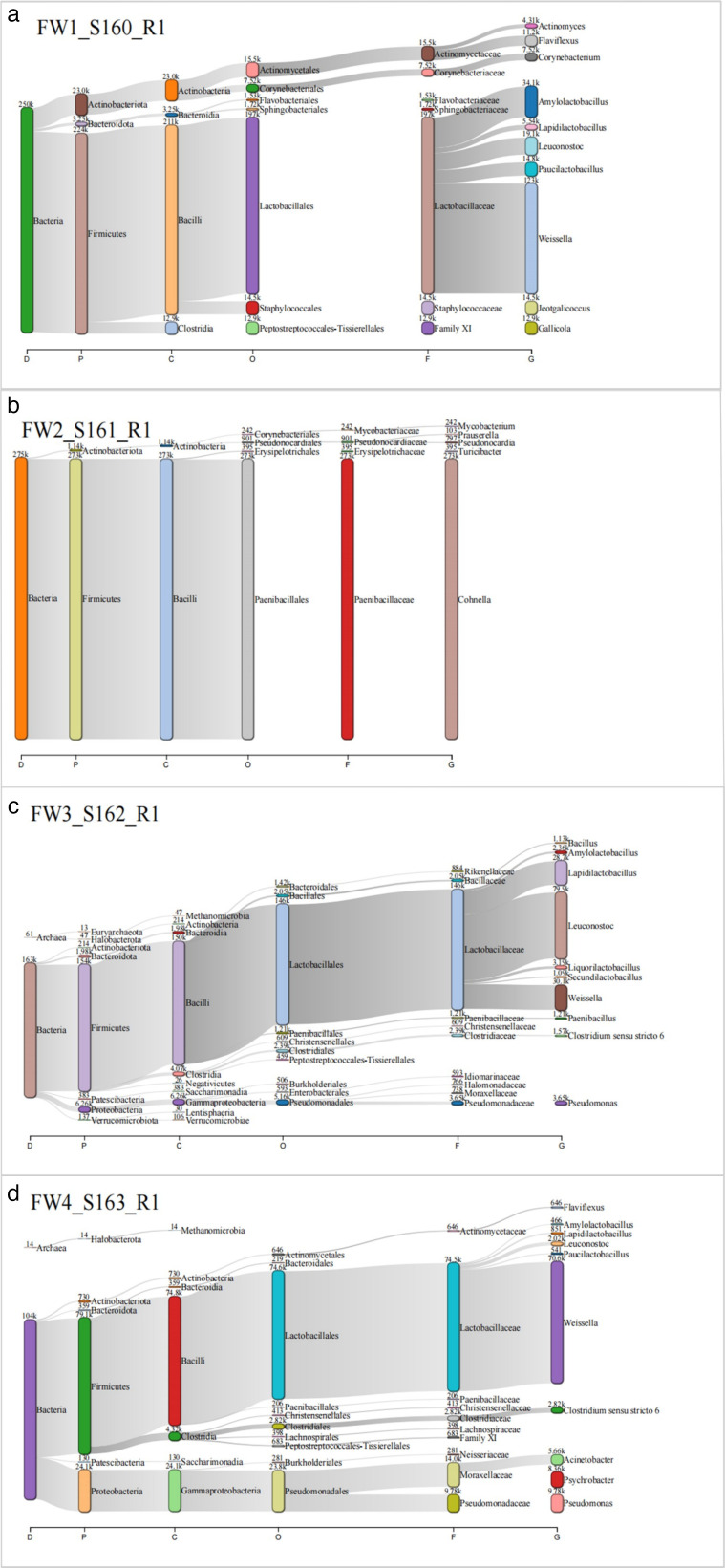
(**a**) Sankey representation of the microbial community of the FW1 sample microbes across the digestion of food waste in the first week. (**b**) Sankey representation of the microbial community of the FW2 sample microbes across the digestion of food waste in the second week. (**c**) Sankey representation of the microbial community of the FW3 sample microbes across the digestion of food waste in the third week. (**d**) Sankey representation of the microbial community of the FW4 sample microbes across the digestion of food waste in the fourth week.

Methanogenic archaea play a crucial role in the final stages of anaerobic digestion by converting VFAs and hydrogen into methane, a key product of AD systems. In the samples analyzed, methanogenic archaea were identified, particularly those from the *Methanobacteria* and *Methanomicrobia* classes. The presence of these methanogens in samples, such as FW3_S162_R1 and FW4_S163_R1, is indicative of their involvement in the methane-producing phase of digestion. The methanogens observed belong to well-known genera, such as *Methanobacterium, Methanoculleus*, and *Methanosarcina*, which are often reported in literature on anaerobic digestion of food waste. *Methanobacterium* species are particularly efficient in hydrogenotrophic methanogenesis, utilizing hydrogen and carbon dioxide to produce methane (39). *Methanosarcina*, a genus capable of both hydrogenotrophic and acetoclastic methanogenesis, also plays an important role in the methane production (40). The presence of these methanogens aligns with findings in previous studies that have characterized methanogenic communities in anaerobic digesters. For example (28), highlighted the dominance *of Methanosarcina* and *Methanobacterium* in food waste digestion, which corresponds with the stages of methane formation and indicates the stability of the AD process. Similarly, Khan et al. (41) reported the growth of hydrogenotrophic and acetoclastic methanogens during the later stages of anaerobic digestion, further supporting the role of these archaea in methane production. The data in Fig. 5a through d show trends in microbial activity across four time points (weeks 1 to 4), revealing how the microbial community adapts as the digestion process progresses.

**Fig 6:**
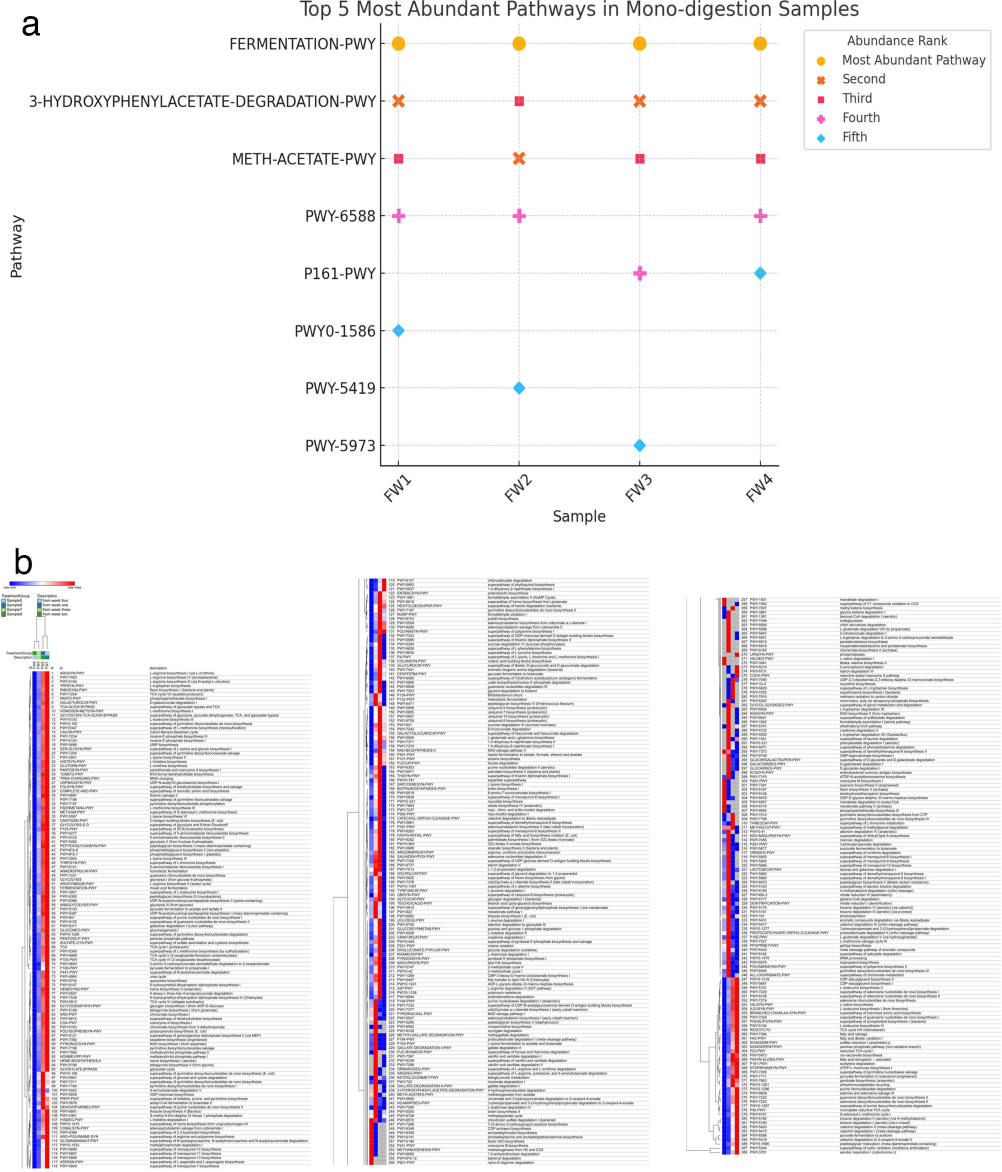
(**a**) Distribution of the five most abundant functional pathways in mono-digestion. (**b**) Functional metabolic pathways in the anaerobic process.

Figure 6a shows the top five most abundant pathways identified in the AD of food waste samples, while Fig. 6b shows the complete profile of all metabolic pathways implicated in the study. This provides insight into the microbial metabolic processes occurring throughout the anaerobic digestion of food waste. A dominant pathway observed throughout all time points is fermentation, represented by the pathway FERMENTATION-PWY (Alcohol Dehydrogenase) with EC number EC 1.1.1.1. This is expected, as fermentation is the primary process in the early stages of anaerobic digestion, where microbes break down complex substrates like carbohydrates, proteins, and lipids into simpler molecules, such as short-chain fatty acids and gases. These metabolites are essential intermediates for further microbial processes, including methanogenesis. In particular, fermentative bacteria like *Clostridia, Lactobacillus*, and *Bacteroides* dominate the early digestion process, breaking down organic compounds into VFAs, which then serve as substrates for methanogens in later stages (33, 42). The continuous abundance of FERMENTATION-PWY throughout the time points highlights its role in breaking down complex organic material into more digestible forms.

The second most abundant pathway across all samples is the 3-hydroxyphenylacetate degradation pathway 3-HYDROXYPHENYLACETATE-DEGRADATION-PWY (with the enzyme 3-hydroxyphenylacetate 3,4-dioxygenase (HpaA/HpaB EC 1.13.11.15). This pathway is involved in the breakdown of aromatic compounds, which are often found in food waste, especially in lignocellulosic materials. The presence of this pathway indicates that the microbial community in the digester is not only capable of digesting simpler substrates but is also effectively degrading more complex, recalcitrant aromatic compounds into smaller, fermentable products. Studies have shown that microbes in anaerobic digestion systems are particularly adept at breaking down such aromatic compounds, which is a key feature in improving the overall efficiency of digestion (28). The ability to metabolize aromatic compounds enhances the efficiency of biogas production, making this a crucial pathway in the process.

The third most abundant pathway, METH-ACETATE-PWY, represents the methanogenesis process, where acetate is converted into methane. This pathway becomes increasingly significant over time, particularly in the later weeks of digestion. The rising abundance of this pathway in weeks 3 and 4 aligns with the well-known shift in anaerobic digestion systems, where the fermentation products (such as acetic acid and hydrogen) are eventually converted to methane by methanogenic archaea. Methanogens, such as *Methanobrevibacter,* are key players in this phase, converting volatile fatty acids like acetate into methane, which is the primary product of biogas (27). As food waste is digested, acetoclastic methanogenesis becomes the dominant pathway for methane production, supporting the importance of this pathway in the later stages of anaerobic digestion (28, 43).

The other pathways, PWY-6588, P161-PWY, PWY0-1586, PWY-5419, and PWY-5973, represent secondary metabolic processes that support the overall breakdown of food waste. These pathways likely involve the fermentation and further breakdown of intermediate metabolites, including the conversion of VFAs and alcohols into methane precursors, as well as the processing of other byproducts generated during the digestion process. Although these pathways are not as abundant as fermentation and methanogenesis, their presence reflects the metabolic flexibility of the microbial community, which can adapt to utilize a wide range of substrates in the digester. Their relatively lower abundance could indicate that they are involved in more specialized or less dominant metabolic processes during digestion (43).

The microbial communities involved in the anaerobic digestion of food waste exhibit distinct characteristics that significantly influence biogas production and process stability. In food waste mono-digestion, the microbial community mainly consists of bacteria, such as *Clostridium, Bacteroides*, and members of the *Lactobacillaceae* family, which are effective at breaking down carbohydrates and simple proteins (44). Additionally, hydrogenotrophic methanogens like *Methanoculleus bourgensis* and *Methanothermobacter* are common, supporting efficient methane production (45). The high carbohydrate content in food waste encourages the growth of fermentative bacteria, resulting in rapid substrate hydrolysis and increased biogas yields (46). The metagenomic analysis in this study supports these findings, emphasizing the enrichment of pathways involved in breaking down carbohydrates, proteins, and lipids that are specific to the digestion process.

Metagenomic profiling of microbial communities involved in the anaerobic digestion of food waste provides valuable insights for improving waste valorization. By identifying dominant taxa and functional pathways responsible for breaking down carbohydrates, proteins, and lipids, this study lays a foundation for optimizing methane production and increasing process stability (47). Such targeted strategies are essential for scaling biogas technologies as a practical renewable energy source. The nutrient-rich digestate produced during digestion can be repurposed as biofertilizer, improving soil fertility and lowering reliance on chemical fertilizers (48). Furthermore, understanding microbial metabolism helps to develop methods to recover value-added compounds, such as organic acids or bioactive molecules, from residual streams.

However, several operational challenges still exist. The heterogeneous composition of food waste causes fluctuations that can interfere with microbial activity and may cause process instability (49). Finding the right balance of feedstock ratios, loading rates, and operating conditions is complicated due to changing microbial interactions. To overcome these challenges, several strategies are recommended. Pre-treatment methods—thermal, chemical, or enzymatic—can enhance substrate breakdown and lower inhibitory compounds (50). Using real-time monitoring systems to track ammonia, pH, and volatile fatty acids can help detect process issues early and support adaptive management. Bioaugmentation with selected microbial groups identified in this study could strengthen system resilience and improve breakdown efficiency (51). Post-digestion treatments, such as composting or pasteurization, help reduce pathogen risks and ensure digestate safety (52). Overall, these results demonstrate how metagenomic methods can boost digestion performance and highlight the need for integrated approaches to address technical challenges. Implementing these strategies can promote sustainable waste management, renewable energy production, and nutrient recycling.

### Conclusion

The comparative metagenomics and metabolomics study of food waste anaerobic digestion has provided significant insights into the microbial community dynamics and metabolic pathways involved throughout the digestion process. Throughout the study, microbial shifts were observed, with the dominance of fermentative bacteria in the early stages of digestion, followed by methanogenic archaea as the process progressed. The analysis highlighted key microbial pathways, including fermentation, aromatic compound degradation, and methanogenesis, which contribute to the efficient breakdown of food waste and methane production. The findings underscore the crucial role of microbial specialization and the interdependence of different microbial groups at various stages of the digestion process. By integrating metagenomic and metabolomic data, this study provides a comprehensive understanding of the microbial and metabolic factors that influence the efficiency of food waste anaerobic digestion, contributing valuable knowledge for optimizing biogas production in waste management systems.

## Data Availability

Sequence data are accessible in the NCBI database under accession numbers SAMN50172698, SAMN50172702, SAMN50172699, and SAMN50172697.
